# Synopsis of *Poeciloderrhis* Stål, 1874, with the description of three new species, and a redescription of the male and female of *Poeciloderrhis ferruginea* (Brunner von Wattenwyl, 1865) from southeast Brazil (Blattodea, Blaberidae, Epilamprinae)

**DOI:** 10.3897/zookeys.545.6172

**Published:** 2015-12-14

**Authors:** Leonardo Cardoso de Oliveira da Silva, Sonia Maria Lopes

**Affiliations:** 1 Universidade Federal do Rio de Janeiro/Museu Nacional, São Cristóvão, Rio de Janeiro, Brazil

**Keywords:** Epilamprinae, morphology, *Poeciloderrhis*, taxonomy

## Abstract

*Poeciloderrhis* Stål (1874) was first described without reference to the included species and without morphological details. The genus was described in a key for the American *Epilampra*, which left only Asian-Australian species in the genus. Roth (1970) was the first to separate *Poeciloderrhis* from *Epilampra* with strong morphological characters. In this contribution, three species of *Poeciloderrhis* are described and a new record from Minas Gerais State is added. The genitalia of the female of *Poeciloderrhis ferruginea* are described for the first time. The genital plates were removed after dissection of the posterior part of the abdomen of males and females, using traditional dissection techniques. The parts were dissected on slides containing glycerin for examination under the stereoscopic microscope. Habitus images, and images of the pronotum, head and genitals were obtained with the help of a camera coupled to a stereomicroscope. The studied material is stored in glycerin inside microvials, and is deposited in the collection of the Museu Nacional. Three new species, *Poeciloderrhis caracensis* **sp. n.**, *Poeciloderrhis minoris* **sp. n.** and *Poeciloderrhis tijucana* **sp. n.** are described and compared with the holotypes of previously described species, together with a redescription of the male and female of *Poeciloderrhis ferruginea* (Brunner von Wattenwyl, 1865).

## Introduction

*Poeciloderrhis* Stål (1874) is a genus that was insufficiently described. The author did not name its constituent species or a description of their morphological details (Stål 1874). The genus was created to separate species of *Epilampra* Burmeister, 1838 that occurred in the Americas from those occurring in Asian and Australian regions.

Kirby (1903) allocated one Australian species to *Heterolampra*, while maintaining *Epilampra* for the American species. He designated *Epilampra brasiliensis* as the type species of *Epilampra* and *Epilampra verticalis* as the type of *Poeciloderrhis*, defining those (*Poeciloderrhis*) geographically as individuals from South America. Shelford (1910) and Princis (1967) placed *Poeciloderrhis* as a synonym of *Epilampra*. However, according to Roth (1970), the tergal modification in the abdomen of *Epilampra* does indeed distinguish it from *Poeciloderrhis* as these modifications would probably lead to mating incompatibilities between the two groups. Roth (1970) also presented the genitalia of six species he considered as *Poeciloderrhis*.

Roth (1970) separated both genera using a key in which only male characters were employed: the presence (*Poeciloderrhis*) or absence (*Epilampra*) of a tergal modification; apex of the dorsal sclerite of the medio-ventral phallomere (L2d) pointed and solidly fused with the median sclerite (L2vm) (*Poeciloderrhis*) or not fused (*Epilampra*); prepucium membranous and setose (*Epilampra*), or not clearly defined and with dense setae (*Poeciloderrhis*); hook (R2) small and robust, without sub-apical incision (*Poeciloderrhis*) or slender and tapering with subapical incision (*Epilampra*) and left phallomere always (L1) without a tuft of setae and fused sclerotized clefts (*Poeciloderrhis*) or setal tufts sometimes present and cleft not fused (*Epilampra*) These traits characterize the genus *Poeciloderrhis*.

Based in the configuration of the tergal modifications of the abdomen, Roth (1970) defined six species, *Poeciloderrhis catharina* Shelford, 1910, *Poeciloderrhis atriventris* (Saussure, 1895), *Poeciloderrhis ferruginea* (Brunner von Wattenwyl, 1865), *Poeciloderrhis proxima* (Brunner von Wattenwyl, 1865), *Poeciloderrhis verticalis* (Burmeister, 1838) and *Poeciloderrhis cribrosa* (Burmeister, 1838).

Pellens and Grandcolas (2008) cited *Epilampra basistriga* (Walker, 1868) as belonging to *Poeciloderrhis*. However, Roth (1970) had already presented the genital structures of that species, making it clear that its configuration is typical of those belonging to *Epilampra* as the apex of the right phallomere is hooked and very elongated; thus being entirely different from those belonging to *Poeciloderrhis*. Lopes Oliveira and Khouri (2010) revalidated the status of *Epilampra basistriga* based on specimens from the Museu Nacional Collection, confirming Roth (1970), which we reaffirm in this paper.

Beccaloni (2015) catalogued 13 species that occur in Brazil, four of which occur in the southeast: *Poeciloderrhis bicolorata* Rocha e Silva & Lopes, 1977, *Poeciloderrhis boraceiana* Lopes & Oliveira, 2006, *Poeciloderrhis imperialis* Rocha e Silva & Jurberg, 1978, *Poeciloderrhis paulistensis* Lopes & Oliveira, 2006) and one in Argentina (*Poeciloderrhis verticalis* Burmeister, 1838).

Three new species are added to the genus and for the first time, the female genitalia of *Poeciloderrhis ferruginea* are described.

## Materials and methods

The genital plates were removed after dissection of the posterior part of the abdomen, using traditional dissection techniques, as described by Lopes and Oliveira (2000). After analysis, the genital plates and genital pieces were stored in microvials containing glycerin and attached to the respective exemplar, a technique developed by Gurney et al. (1964). The terminology for the genitalia and the taxonomic classification follows Roth (2003). The new species were compared with other known species of *Poeciloderrhis* and species of *Epilampra* and deposited in the Blattodea Collection of the Museu Nacional of Rio de Janeiro (MNRJ). Digital images of the habitus, pronotum, head and genital sclerites were taken with a camera mounted on a stereoscope. The holotypes were deposited in the collection of the Department of Entomology at the Museu Nacional of Rio de Janeiro (MNRJ).

## Results

### Poeciloderrhis ferruginea

Taxon classificationAnimaliaBlattodeaBlaberidae

(Brunner von Wattenwyl, 1865)

Figures 1–10 , 11–16

#### General coloration.

**Figures 1–10.**
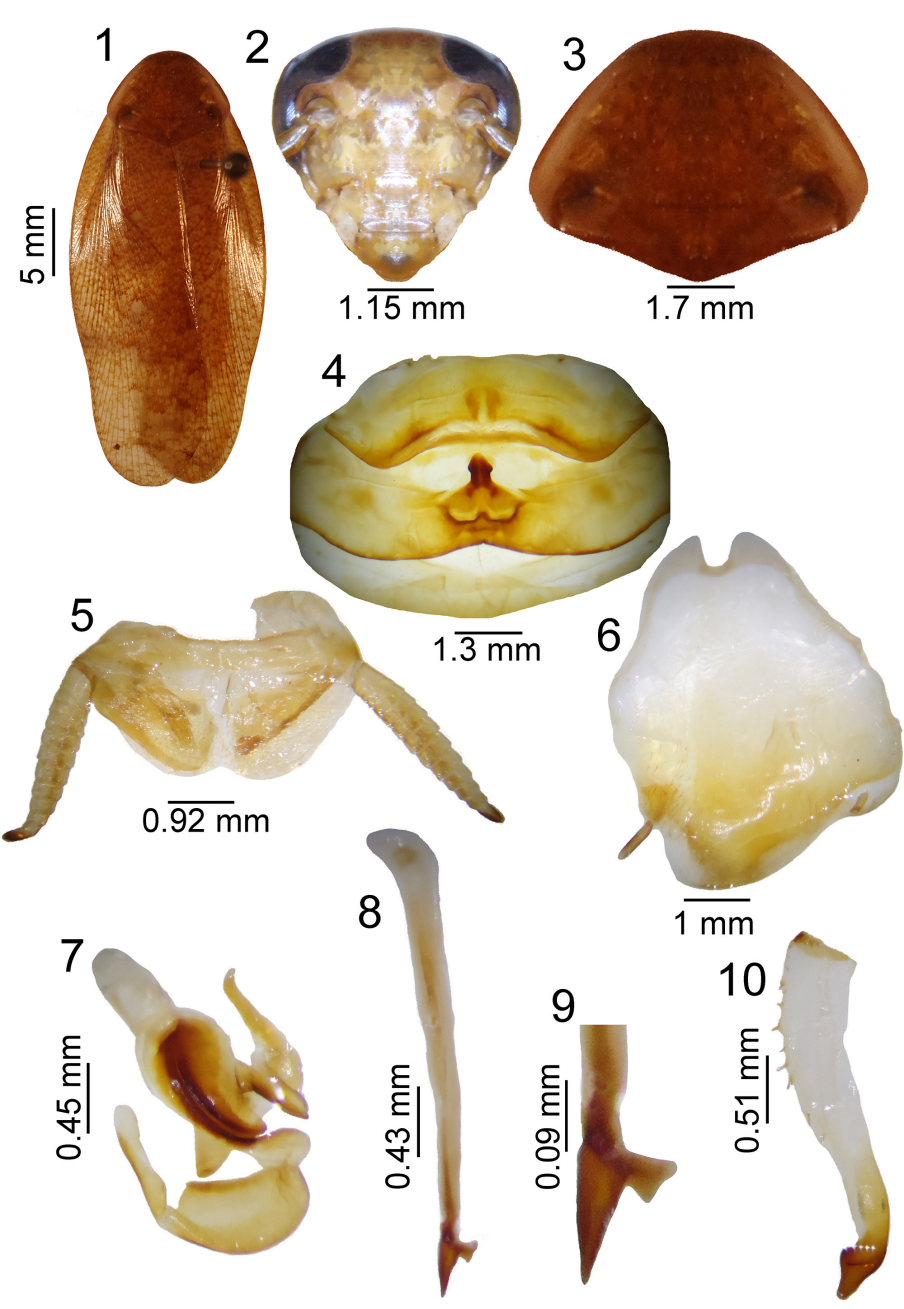
*Poeciloderrhis ferruginea* (Brunner Von Wattenwyl, 1865) male **1** habitus **2** head, ventral view **3** pronotum, dorsal view **4** tergal modification in 1^th ^tergite and 2^th^ **5** supra-anal plate, dorsal view **6** subgenital plate, ventral view **7** left phallomere, dorsal view **8–9** median sclerite, dorsal view **10** right phallomere.

**Figures 11–16.**
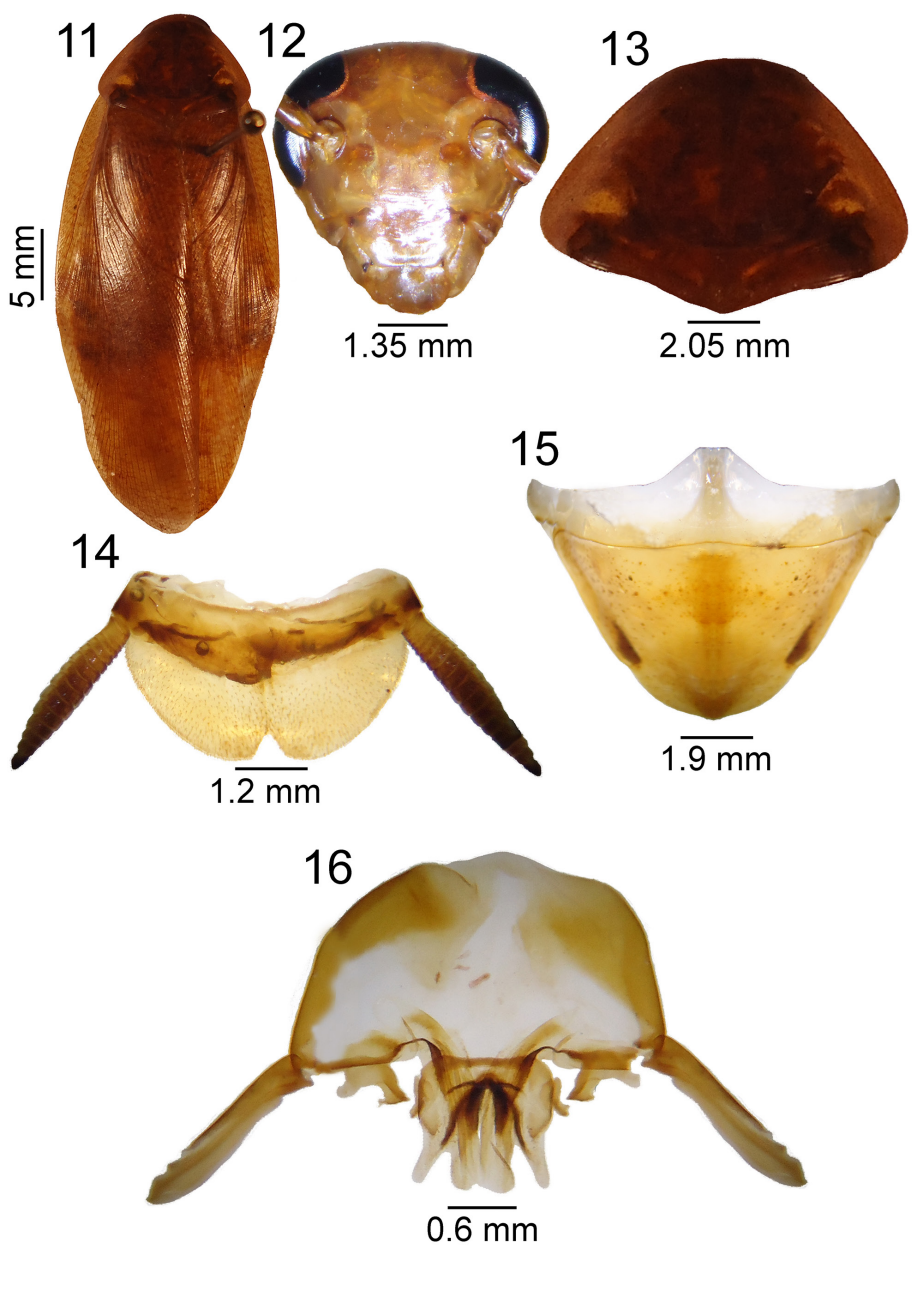
*Poeciloderrhis ferruginea* (Brunner Von Wattenwyl, 1865) female. **11** habitus **12** head, ventral view **13** pronotum, dorsal view **14** supra-anal plate, dorsal view **15** subgenital plate, ventral view; **16** valves, dorsal view.

Shiny dark brown (Figs 1 and 11). Head with vertex, interocular space between ocellar fenestra and antennae brown. Two brown spots below ocellar fenestra. Maxillary palps with brown apical segment, with golden cilia. Eyes black (Fig. 2). Pronotum semi-transparent with dark brown punctuation (Fig. 3). Tegmen semi-transparent, light brown, bearing dark brown spots. Legs light brown with spines, arolia and claws brown. Abdomen light brown with brown punctation.

#### Dimensions (male) (mm).

Total length: 32; total length of pronotum: 6.57; width of pronotum: 7.5; length of tegmen: 27.1; width of tegmen: 6.1.

#### Head.

Triangular with rounded angles, vertex hidden in dorsal view; interocular space measuring about 1 mm; antennae long, slender and tomentose, reaching beyond apex of cerci. Eyes antero-lateral in position; maxillary palps with first and second segments reduced, the second measuring 0.41 mm, third segment 25% longer than fourth and 25% shorter than fifth segment, which is a little more dilated and very tomentose.

#### Thorax.

Pronotum wide, pentagonal, convex, with curved margins, base with small median projection. Legs developed, half of femur I with anteroventral surface bearing four strong spines, followed by series of small spines towards apex, where two strong apical spines are present; posteroventral surface with three strong spines, one apical; femora II and III showing strong spaced spines on their ventral surfaces. Pulvilli present on fourth tarsal segment, claws symmetrical and specialized, with two rows of small spines on ventral surface. Tegmen reaching beyond apex of abdomen. Marginal field wide, slightly concave, convex, curved, discoidal field convex and short at apex, anal field convex and well-marked.

#### Abdomen.

Tergal modification pyramid-shaped, tall, with cilia, located on first tergite and depression followed by curved stalk on second tergite (Fig. 4). Supra-anal plate round with cerci reaching beyond the plate (Fig. 5). Subgenital plate asymmetric, with two slender styles, one long, and one short; larger style with sharper lateral tomentosity (Fig. 6). Left phallomere with median structure shaped as sclerotized cleft (Fig. 7). Median apical sclerite spiniform with intense sclerotization, with membranous sclerotized structure bearing lateral shaft and slender pre-apical structure (Figs 8, 9). Right phallomere with straight apex and a small spine next to apex; lateral portion of phallomere with spines (Fig. 10).

#### Dimensions (female) (mm).

Total length: 33.5; total length of pronotum: 7.7; width of pronotum: 9.45; length of tegmen: 29.3; width of tegmen: 7.8. Female larger than male; brown color of vertex extending to frons (Fig. 12); pronotum brown; in the abdomen, anal plate enlarged with indistinct slit medially, cerci poorly developed reaching apex of plate (Fig. 14); subgenital plate triangular (Fig. 15); female genitalia in three pairs of valves, of which the first is the most developed and widens toward apex, the second is the smallest and most sclerotized, and the third is more slender apically and slightly smaller in length than the first. Valvifers tapered and very small (Fig. 16).

#### Material examined.

1 ♂ Brazil, Minas Gerais, Catas Altas, RPPN Serra da Caraça, collected at 1490 m; 3 to 7/XII/ 2013. J. P. Botero, A. Carelli & G. C. Queiroz cols; 1 ♀ Brazil, data the same as for male.

#### Diagnosis.

The male of *Poeciloderrhis ferruginea* differs from the male in the size of the body, brown color of the vertex extending to the frons; enlarged supra-anal plate with indistinct median indentation, cerci poorly developed reaching apex of the plate; subgenital plate triangular; female genitalia with 3 pairs of valves; valvifers slender and small.

### Poeciloderrhis caracensis sp. n.

Taxon classificationAnimaliaBlattodeaBlaberidae

http://zoobank.org/B6E09FF0-2700-4DE3-982F-DF06205E325E

Figures 17–26

#### General coloration.

**Figures 17–26.**
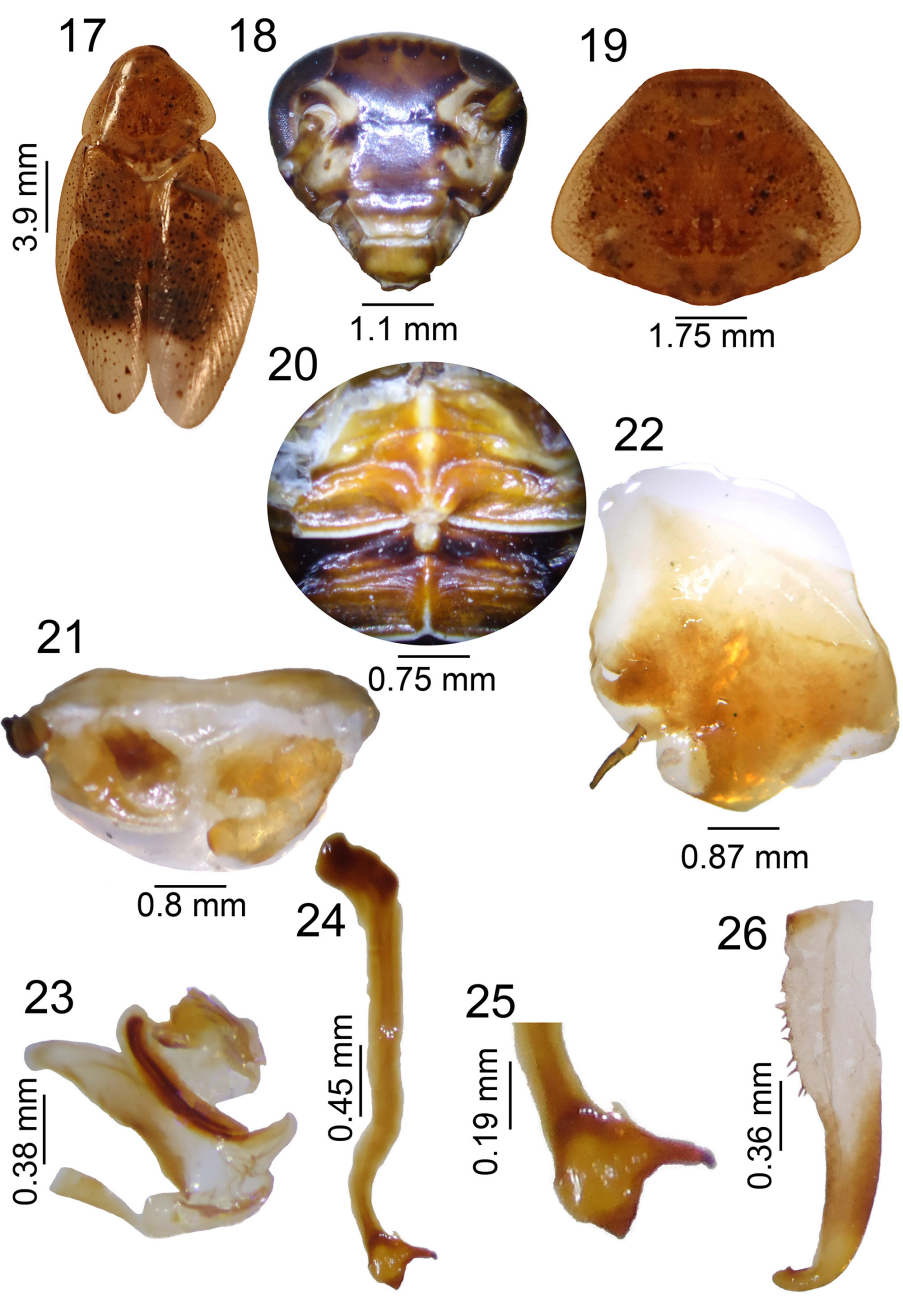
*Poeciloderrhis caracensis* sp. n. male. **17** habitus **18** head, ventral view **19** pronotum, dorsal view **20** tergal modification in 1^st ^tergite and 2^nd^ **21** supra-anal plate, dorsal view **22** subgenital plate, ventral view **23** left phallomere, dorsal view **24–25** median sclerite, dorsal view **26** right phallomere.

Shiny light brown (Fig. 17). Head with apex light brown; ocelli and region above ocelli and below antennal insertions brown. Eyes dark brown (Fig. 18). Pronotum light brown, semi-transparent with dark brown punctations and symmetrical spot centrally (Fig. 19); tegmina light brown, semi-transparent with dark brown spots. Legs light brown and pulvilli with spines, arolia and claws dark brown. Abdomen dark brown.

#### Dimensions (mm).

Total length: 21.3; total length of pronotum: 6.2; width of pronotum: 8.1; length of tegmen: 8.1; width of tegmen: 6.5.

#### Head.

Triangular with rounded edges, vertex slightly exposed in dorsal view; interocular space measuring about 1 mm. Eyes positioned antero-laterally; maxillary palps with first and second segments reduced, the latter 0.45 mm, third segment the largest, 25% longer than the fourth, which is equal to fifth and a little more dilated and very tomentose.

#### Thorax.

Pronotum wide, pentagonal, convex, with curved margins, base bearing small median projection. Legs developed, femur I bearing 3-6 strong spines up to the middle, followed by series of small spines towards apex, where two apical strong spines are present; posteroventral surface with three strong spines, one apical; femora II and III with strong spaced spines on ventral surfaces. Pulvilli present on four tarsal segments, claws symmetrical and specialized, with two rows of small spines on ventral surface, similarly to those of legs. Tegmen not exceeding apex of abdomen.

#### Abdomen.

Tergal modification a tall structure on second segment, first segment with three median humps (Fig. 20). Supra-anal plate round with cerci short and tomentose dorsally (Fig. 21). Subgenital plate asymmetric, with left style in ventral view slender, filiform and well sclerotized; right style absent (Fig. 22). Genitalia with left phallomere bearing median structure shaped as sclerotized cleft (Fig. 23); median sclerite with quadrangular apex and a curved, well-developed spine (Fig. 24, 25). Right phallomere with curved apex bearing one small spine; prepucium with spines (Fig. 26).

#### Material examined.

Holotype ♂, Brazil, Minas Gerais, Serra do Caraça, XI/1969. F. M. Oliveira col. Paratype ♂ Brazil, Mato Grosso, Sinop, X/1974, Alvarenga & Roppa col. Paratype ♂ without locality data.

#### Diagnosis.

*Poeciloderrhis caracensis* sp. n. is similar to *Poeciloderrhis verticalis* (Burmeister, 1838) in coloration and habitus (in Roth, 1970 fig. 50), but differs in size (*Poeciloderrhis verticalis* is 38,8 mm). *Poeciloderrhis caracensis* sp. n. also differs from *Poeciloderrhis santosi* (Rocha e Silva & Lopes, 1976) in size (*Poeciloderrhis santosi* is 19,6 mm). The tergal modification also differentiates *Poeciloderrhis verticalis* and *Poeciloderrhis caracensis*. In *Poeciloderrhis verticalis* the first segment has one raised medial ridge and in the second segment has two projections; one hooked (basal) and one pyramidal (apical).

#### Etymology.

The species epithet refers to the locality where it was collected.

### Poeciloderrhis minoris sp. n.

Taxon classificationAnimaliaBlattodeaBlaberidae

http://zoobank.org/FAEB84A1-9D28-444A-9DF6-123F794BE25C

Figures 27–36

#### General coloration.

**Figures 27–36.**
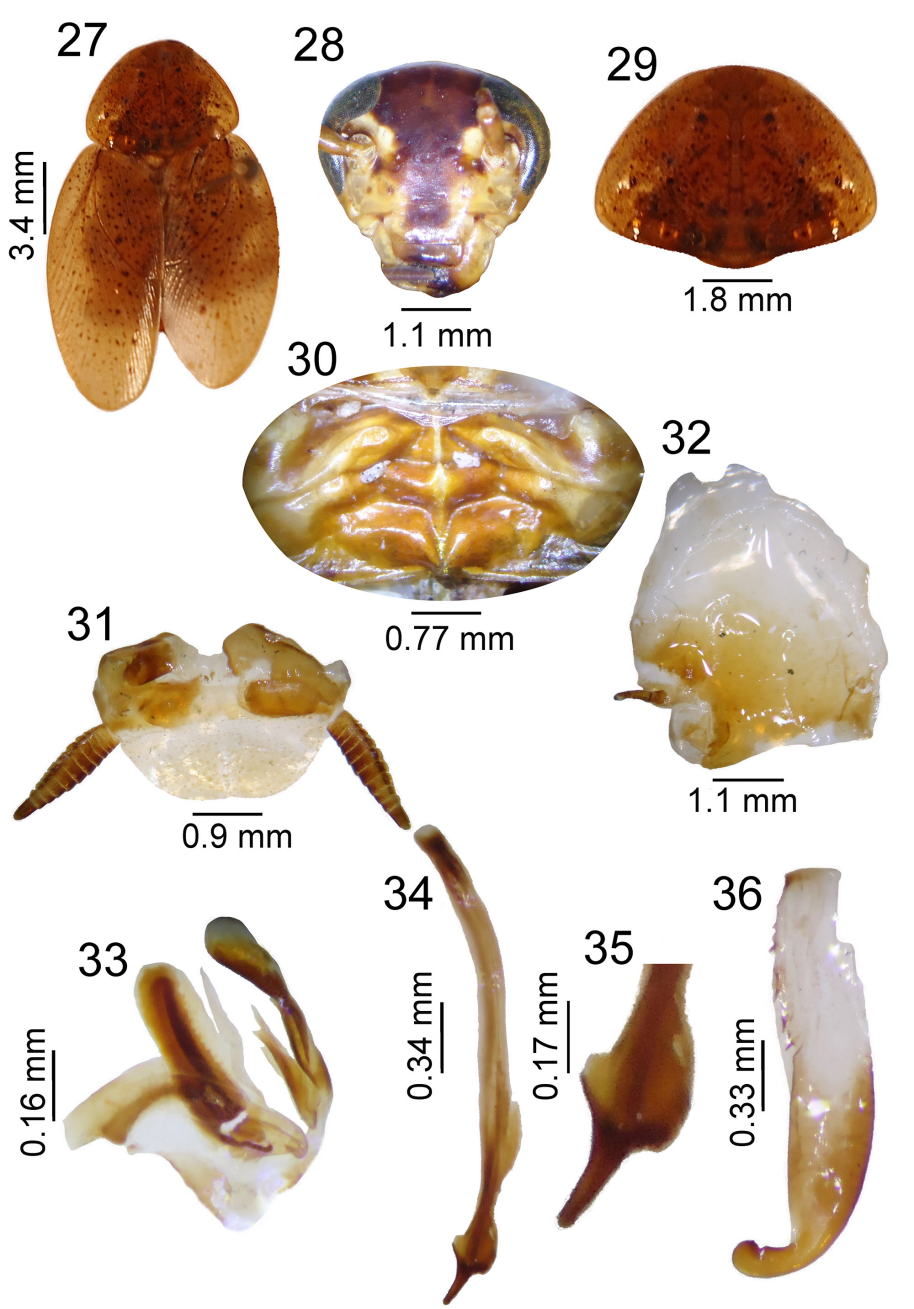
*Poeciloderrhis minoris* sp. n. male. **27** habitus **28** head, ventral view **29** pronotum, dorsal view **30** tergal modification in 1^st ^tergite and 2^nd^ **31** supra-anal plate, dorsal view **32** subgenital plate, ventral view **33** left phallomere, dorsal view **34–35** median sclerite, dorsal view **36** right phallomere.

Shiny light brown (Fig. 27). Head with vertex light brown; interocular space between ocelli and antennal insertions brown; other regions light brown with brown stripe from between antennae to the tip of the clypeus. Ocelli brown; maxillary palps with apical segment brown, cilia golden. Eye black (Fig. 28). Pronotum light brown, semi-transparent with dark brown punctations (Fig. 29); tegmen light brown, semi-transparent with dark brown spots. Legs light brown with spine, pulvilli, arolia and claws dark brown. Abdomen light brown with brown punctations.

#### Dimensions (mm).

Total length: 17.1; total length of pronotum: 5.4; width of pronotum: 7.1; length of tegmen: 12.8; width of tegmen: 5.3.

#### Head.

Triangular with rounded edges, vertex slightly exposed in dorsal view; interocular space 1.4 mm. Eyes positioned antero-laterally; maxillary palps with first and second segments reduced, the latter 0.43 mm, third segment 25% of fourth, and smaller than fifth segment, which is a little more dilated and very tomentose.

#### Thorax.

Pronotum wide, pentagonal, convex, with curved margins, base bearing small median projection. Legs developed; proximal half of femur I bearing 5 strong spines on anteroventral surface followed by series of small spines towards apex, where two strong apical spines are present; posteroventral surface with three strong spines, one apical; femora II and III with strong spaced spines on ventral surfaces. Pulvilli present on four tarsal segments, claws symmetrical and specialized, with two rows of small spines on ventral surface. Tegmen not reaching beyond apex of abdomen.

#### Abdomen.

Tergal modification a tall triangular structure between two humps. Below that, a pyramid-shaped hump (Fig. 30). Supra-anal plate round with cerci short; cerci with row of cilia on each segment (Fig. 31). Subgenital plate asymmetrical with apical cleft, with left style in dorsal view long, filiform and sclerotized; right style absent (Fig. 32). Internal genitalia with left phallomere with median structure shaped as sclerotized cleft (Fig. 33); median sclerite developed as a spine with sclerotized apical curvature (Figs 34, 35). Right phallomere with curved apex and a small thorn near the apex; lateral shaft of phallomere with spines (Fig. 36)

#### Material examined.

Holotype ♂, Brazil, São Paulo, Campos do Jordão, X/1954. P.H. Saldanha col.

#### Diagnosis.

*Poeciloderrhis minoris* sp. n. is small (17.1 mm) when compared with the other described species. It has short wings, similar to the wings of *Poeciloderrhis santosi* (Rocha e Silva & Lopes, 1976), but can be distinguished from it in the tergal modification that is similar to *Poeciloderrhis caracensis* sp. n., general coloration and genital parts: the right phallomere is wider apically and the apical thorn at the apex of the median sclerite is reduced.

#### Etymology.

The *minoris* is from the Latin minor, referring to the small size (17.1 mm) of this species when compared to the other described species.

### Poeciloderrhis tijucana sp. n.

Taxon classificationAnimaliaBlattodeaBlaberidae

http://zoobank.org/F58D23FE-B4D5-4595-A7A6-657E9318E3F0

Figures 37–45

#### General coloration.

**Figures 37–46.**
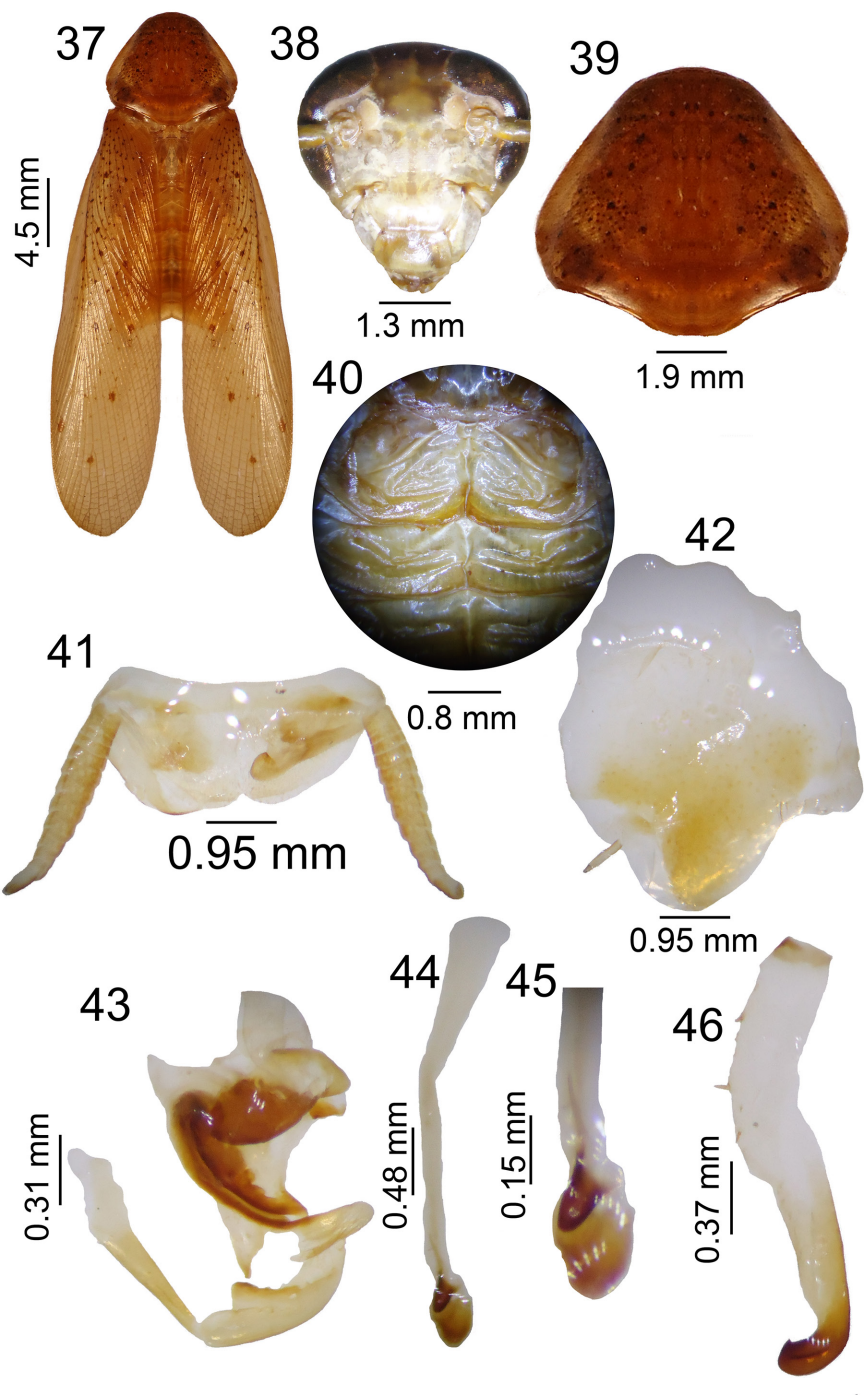
*Poeciloderrhis tijucana* sp. n. male **37** habitus; **38** head, ventral view; **39** pronotum, dorsal view; **40** tergal modification in 1^st ^tergite and 2^nd^; **41** supra-anal plate, dorsal view; **42** subgenital plate, ventral view; **43** left phallomere, dorsal view; **44–45** median sclerite, dorsal view; **46** right phallomere.

Shiny light brown (Fig. 37). Head with vertex, interocular space between ocelli brown; other regions light brown. Maxillary palps with apical segment brown, cilia golden. Eye brown (Fig. 38). Pronotum light brown, with dark brown punctations (Fig. 39); tegmen light brown, with dark brown punctations. Legs light brown with spine, pulvilli, arolia and claws dark brown. Abdomen light brown.

#### Dimensions (mm).

Total length: 33; total length of pronotum: 7.0; width of pronotum: 8.25; length of tegmen: 28; width of tegmen: 7.9.

#### Head.

Triangular with rounded edges, vertex slightly exposed; interocular space about 1.0 mm. Antennae long, slender, and tomentose, reaching beyond apex of cerci. Eyes positioned antero-laterally; maxillary palps with first and second segments reduced, the latter 0.46 mm, third segment 25% larger than fourth and 10% larger than fifth, which is a little more dilated and very tomentose.

#### Thorax.

Pronotum ample, pentagonal, convex, with curved margins, base bearing small median projection. Legs developed, femur I bearing 7 strong spines on anteroventral half, followed by series of small spines towards apex, where two strong apical spines are present; posteroventral surface with three strong spines, one apical; femora II and III with strong spaced spines on ventral surfaces. Pulvilli present on four tarsal segments, claws symmetrical and specialized, with two rows of small spines on ventral surface. Tegmen not reaching beyond apex of abdomen.

#### Abdomen.

Tergal modification with two latero-apical humps converging toward thorax on first segment and small median hump on second tergite (Fig. 40). Supra-anal plate round with cerci reaching over apex of plate and short apical cleft (Fig. 41). Subgenital plate asymmetrical with apical cleft, with one long, filiform style and another inconspicuous one (Fig. 42). Genitalia with left phallomere with median structure shaped as sclerotized cleft (Fig. 43); median sclerite round apically and sclerotized on pre-apical region, with prepucium membranous bearing small spines (Fig. 44, 45). Right phallomere with curved apex and a small apical spine (Fig. 46).

#### Material examined.

Holotype ♂, Brazil, Rio de Janeiro, Tijuca. No date and collector information.

#### Etymology.

This species is named after the type locality, Tijuca, in Rio de Janeiro.

#### Diagnosis.

This species is close to *Poeciloderrhis ferruginea* in size, differing in: the coloration of the tegmina being more intense in *Poeciloderrhis ferruginea*, ventral view, the subgenital plate having the right style small but distinct in *Poeciloderrhis ferruginea*, and in the median sclerite having a sharp apex in *Poeciloderrhis ferruginea*.

## Supplementary Material

XML Treatment for Poeciloderrhis ferruginea

XML Treatment for Poeciloderrhis caracensis

XML Treatment for Poeciloderrhis minoris

XML Treatment for Poeciloderrhis tijucana
